# *IDH1* and *IDH2* mutations in lung adenocarcinomas: Evidences of subclonal evolution

**DOI:** 10.1002/cam4.3058

**Published:** 2020-04-25

**Authors:** Erika F. Rodriguez, Federico De Marchi, Parvez M. Lokhandwala, Deborah Belchis, Rena Xian, Christopher D. Gocke, James R. Eshleman, Peter Illei, Ming‐Tseh Li

**Affiliations:** ^1^ Department of Pathology Johns Hopkins University School of Medicine Baltimore MD USA; ^2^ Department of Oncology Johns Hopkins University School of Medicine Baltimore MD USA

**Keywords:** cytosine deamination, *IDH1*, *IDH2*, lung cancers, parallel evolution

## Abstract

**Background:**

Selective IDH1 and IDH2 inhibitors have been approved for targeted therapy of acute myeloid leukemia. Clinical trials for solid tumors with *IDH1* and *IDH2* (*IDH1*/*2*) mutations are ongoing. Reports of *IDH1*/*2*‐mutated non–small cell lung cancers (NSCLCs), however, are limited.

**Methods:**

We evaluated *IDH1*/*2* mutations in 1,924 NSCLC specimens (92% adenocarcinoma) using a next‐generation sequencing assay.

**Results:**

Retrospective quality assessments identified false detection of *IDH1* c.395G>A (p.R132H) resulting from cytosine deamination (C:G→T:A) artifact in one specimen. *IDH1*/*2* mutations were detected in 9 (0.5%) adenocarcinomas taken by fine‐needle aspiration (n = 3), thoracentesis (n = 2) or core biopsy (n = 4). All nine adenocarcinomas showed high‐grade features. Extensive clear cell change, however, was not observed. High expression (50% or greater) of PD‐L1 was observed in two of five specimens examined. *IDH1*/*2* mutations were associated with old age, smoking history, and coexisting *KRAS* mutation. Lower than expected variant allele frequency of *IDH1*/*2* mutants and coexistence of *IDH1/2* mutations with known trunk drivers in the *BRAF*, *EGFR,* and *KRAS* genes suggest they could be branching drivers leading to subclonal evolution in lung adenocarcinomas. Multiregional analysis of an adenocarcinoma harboring two *IDH2* mutations revealed parallel evolution originating from a *KRAS*‐mutated lineage, further supporting subclonal evolution promoted by *IDH1*/*2* mutations.

**Conclusions:**

*IDH1*/*2* mutations in NSCLCs are uncommon. They occur in adenocarcinomas with high‐grade features and may be branching drivers leading to subclonal evolution. Accumulation of more *IDH1*/*2*‐mutated NSCLCs is needed to clarify their clinicopathological characteristics and implications for targeted therapy.

## Graphical abstract

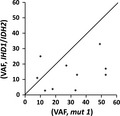

## 1 INTRODUCTION

Mutational profiling identifies genomic alterations for targeted therapy in metastatic non–small cell lung cancers (NSCLCs). Several targeted therapeutic agents have been approved for metastatic NSCLCs with *EGFR* mutations, *BRAF* p.V600E, *ALK* translocations, and *ROS1* translocations.^1^, ^2^ Mutational profiling of these genomic alterations is considered standard of care for patients with metastatic NSCLCs.^3^ Integrated multiplatform analyses including whole‐exon sequencing and whole‐genome sequencing have uncovered additional genomic alterations in NSCLCs with potential implications for targeted therapy, such as *ERBB2* mutations, *MET* mutations and translocations of the *RET*, *NTRK1*, *NTRK2,* and *NTRK3* genes.^1^, ^2^

*IDH1* mutations involving codon 132 and *IDH2* mutations involving codons 140 and 172 occur in a variety of human cancers, including acute myeloid leukemia (AML), diffuse gliomas, cholangiocarcinoma, and chondrosarcoma.^4^, ^5^, ^6^, ^7^, ^8^, ^9^, ^10^, ^11^ *IDH1* and *IDH2* (*IDH1*/*2*) mutations were also reported in NSCLCs with a much lower incidence (0.4%‐1.1%).^12^, ^13^, ^14^ *IDH1*/*2* mutants lead to accumulation of D‐2‐hydroxyglutarate through neoenzymatic conversion, and subsequent oncogenic effects including epigenetic alterations.^15^, ^16^ IDH2 inhibitor (Enasidenib or AG‐221) and IDH1 inhibitor (Ivosidenib or AG‐120) have been approved by the Food and Drug Administration in the United States for targeted therapy of AML.^6^, ^7^ Several clinical trials of IDH1/2 inhibitors for advanced solid tumors, such as NCT02073994 (AG‐120 for *IDH1* mutations), NCT02746081 (BAY1436032 for *IDH1* mutations), and NCT02481154 (AG‐881 for *IDH1*/*2* mutations) are ongoing. Clinical pharmacokinetics and pharmacodynamics studies have shown robust and persistent inhibition of plasma D‐2‐hydroxyglutarate by oral ivosidenib.^17^

In this study for quality assessment, next‐generation sequencing (NGS) was examined in a large cohort of NSCLC specimens to elucidate the incidence of *IDH1*/*2* mutations and the clinicopathological and molecular characteristics of *IDH1*/*2*‐mutated NSCLCs.

## 2 MATERIALS AND METHODS

### 2.1 Materials

NGS results from 1924 lung cancer specimens (1778 adenocarcinomas, 24 adenosquamous carcinomas, 12 adenocarcinomas in situ, and 110 NSCLCs) submitted between April 2013 and December 2018 were analyzed for mutations in *IDH1* and *IDH2* genes. For multiple specimens taken from the same tumor (such as biopsy and resection specimens, or primary and metastatic tumor specimens) and showing an identical mutation status, only one specimen was included. Specimens with prior EGFR tyrosine kinase inhibitor therapy were also excluded. Accompanied hematoxylin and eosin–stained slides were reviewed by a pulmonary pathologist (PI) and/or a molecular pathologist (MTL). DNA was isolated from formalin‐fixed paraffin‐embedded (FFPE) tissues using Pinpoint reagents (ZymoResearch) and purified using QIAmp DNA kit (Qiagen) as described previously.^18^ After April 2017, DNA was isolated from FFPE tissues using Tissue Preparation System (Siemens) according the manufacturer's protocol. Concentration of DNA was determined by Qubit 2.0 Fluorometer (Life Technologies). The Johns Hopkins Institutional Review Board granted approval to this study.

### 2.2 Next‐generation sequencing (NGS)

NGS was conducted using AmpliSeq Cancer Hotspot Panel (v2) (Life Technologies) for targeted multigene amplification, as described previously.^18^, ^19^ Mutations were identified and annotated through both Torrent Variant Caller (Life Technologies) and direct visual inspection of the binary sequence alignment/map file using the Broad Institute's Integrative Genomics Viewer (IGV) (http://www.broadinstitute.org/igv/) as described previously.^20^ In addition to *IDH1* (NM_005896) and *IDH2* (NM_002168), mutations in the *AKT1* (NM_005163), *BRAF* (NM_004333), *EGFR* (NM_005228), *ERBB2* (NM_004448), *KRAS* (NM_033360), *NRAS* (NM_002524), and *PIK3CA* (NM_006218) genes were analyzed for each specimen. The analytic performance characteristics of this assay for lung cancers have been reported previously.^19^ During our validation of this NGS assay, a cutoff of background noise at 2% was chosen for single‐nucleotide variations.^21^

### 2.3 Immunohistochemical stains

Immunochemical stains for TTF1, Napsin A, and programed death ligand 1 (PD‐L1) were performed as routine clinical assays using Ventana XT (Ventana Medical Systems) and Leica Bond III (Leica Microsystems) automated immunohistochemistry platform as described previously.^22^ The monoclonal antibody clone 22C3 (KEYTRUDA) (Neogenomics) and OptiView Detection System (Ventana Medical Systems) were used for PD‐L1 staining. High expression is defined as 50% or greater Tumor Proportion Score.

#### 2.3.1 Statistical analysis

The Fisher exact test or χ^2^ test was performed to calculate *P* values.

## 3 RESULTS

### 3.1 *IDH1*/*2* mutations in lung adenocarcinomas

**FIGURE 1:**
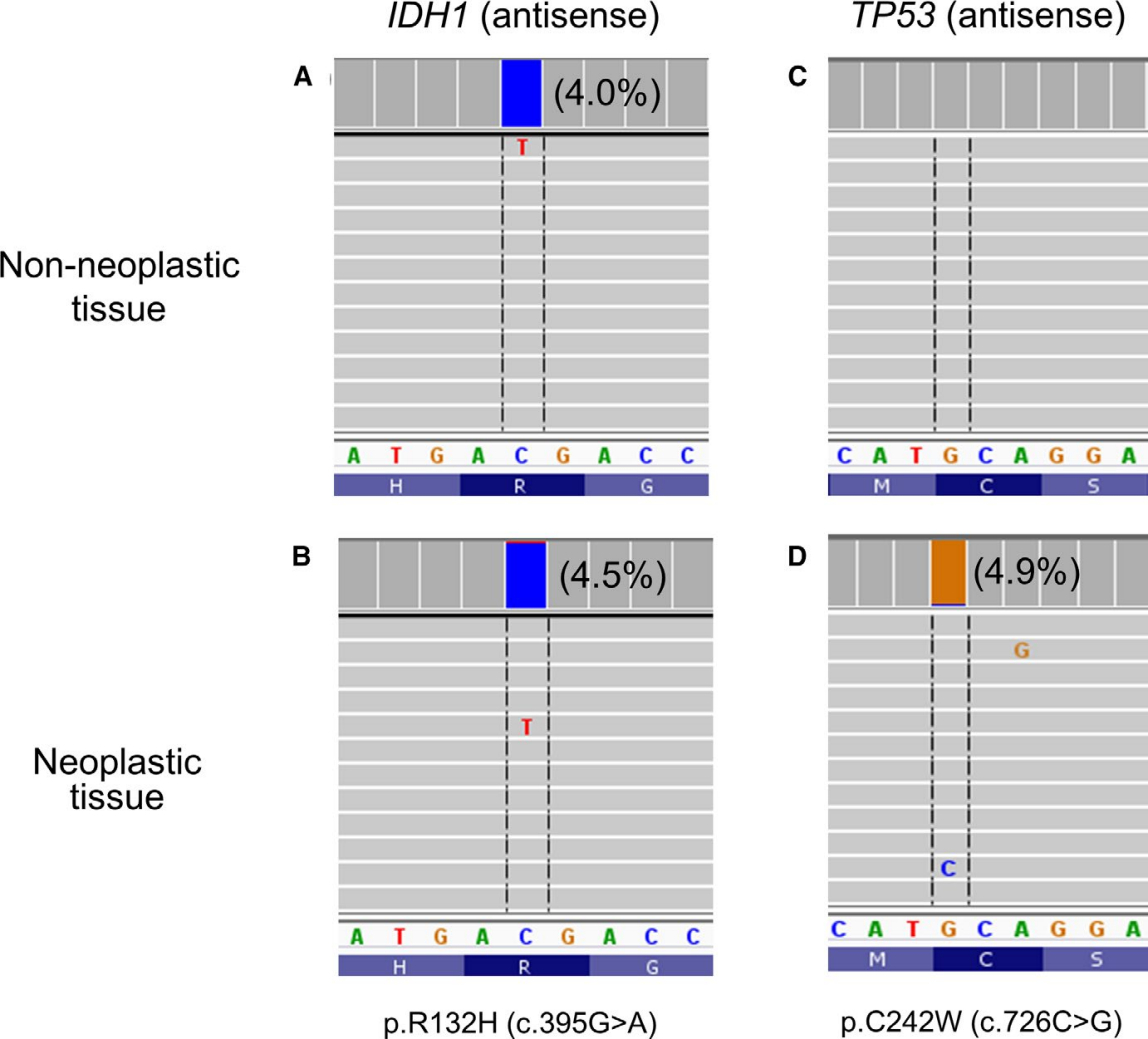
False detection of IDH1 p.R132H (c.395G>A) change resulting from cytosine deamination artifact. In case 10, IDH1 c.395G>A was detected in the non‐neoplastic subarea (A) and neoplastic subarea (B). TP53 c.726C>G was not detected in the non‐neoplastic subarea (2/3792 = 0.05%, below the limit of detection) (C), but was present in the neoplastic subarea (D). Percentage in parentheses indicates variant allele frequency

**FIGURE 2:**
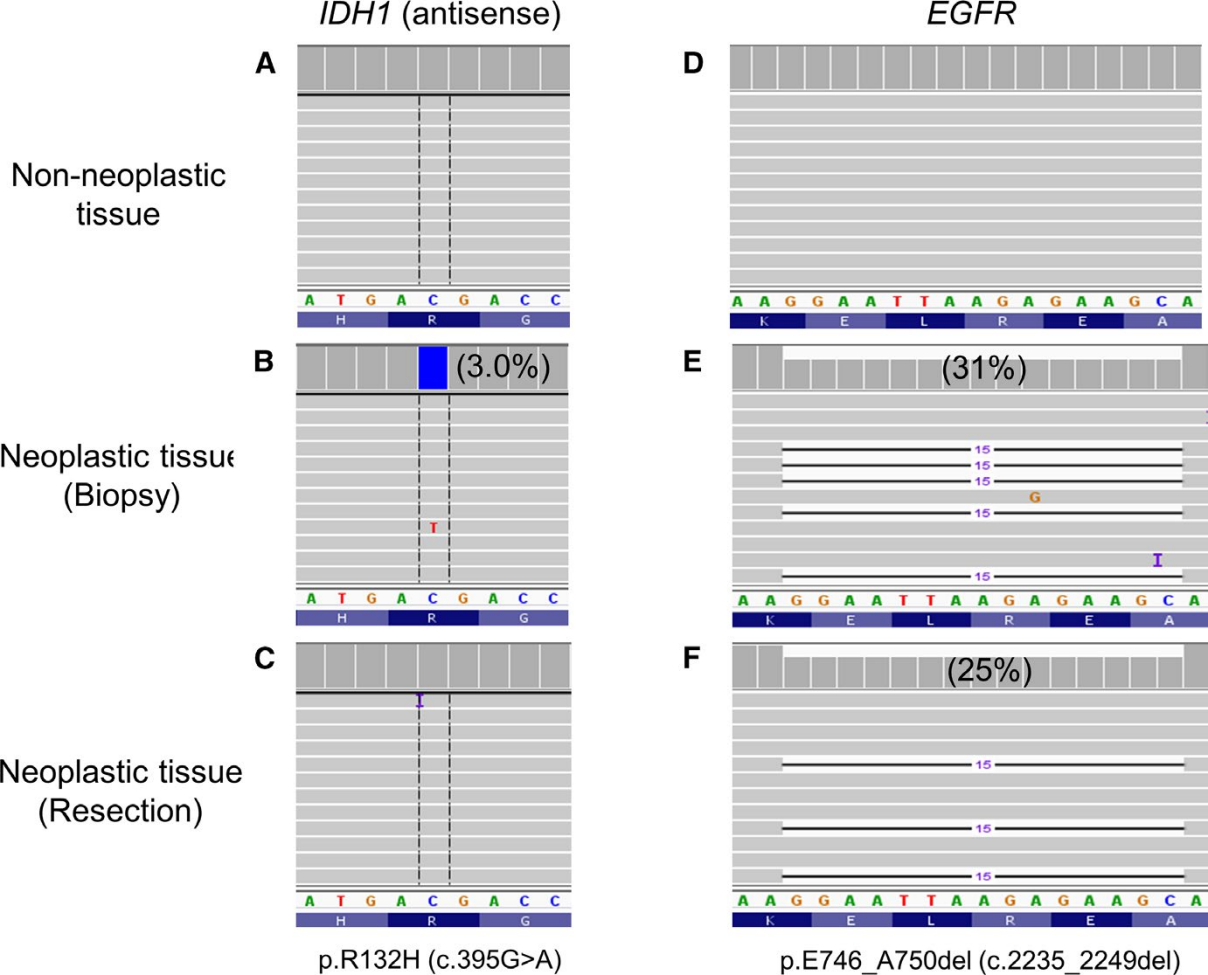
Subclonal evolution of IDH1 p.R132H (c.395G>A) mutation. In case 3, NGS revealed absence of IDH1 (7/3344 = 0.2%, below the limit of detection) and EGFR mutations in the non‐neoplastic tissue (A and D), 3.0% IDH1 and 31% EGFR mutations in the neoplastic tissue of biopsy specimen (B and E), and only EGFR mutation in the neoplastic tissue of resection specimen (6/5592 = 0.1% IDH1, below the limit of detection) (C and F). Percentage in parentheses indicates variant allele frequency

NGS detected 11 *IDH1*/*2* mutations in 10 specimens (Table 1). These included 8 specimens with an *IDH1* mutation (4 with p.R132L or c.395G>T, 3 with p.R132H or c.395G>A, 1 with p.R132G or c.394C>G), 1 specimen with an *IDH2* mutation (p. R172S or c.516G>T), and 1 specimen with 2 *IHD2* mutations (p.R140Q or c.419G>A and p.R172M or c.515G>T). The variant allele frequency (VAF) was less than 5% in 4 specimens, including cases 3 and 10 harboring a cytosine deamination change (p.R132H or c.395G>A). Repeat of NGS showed a concordant VAF (2.9% p.R132H in case 3, 3.3% p.R132G in case 5, 3.5% p.R132L in case 6, and 4.5% p.R132H in case 10). DNA was isolated from the adjacent nonneoplastic tissues of case 3 (one subarea with no tumors) and case 10 (two separate subareas with no tumors). NGS showed c.395G>A in both nonneoplastic and neoplastic subareas of case 10 (Figure 1A,B), but only in the neoplastic subarea of case 3 (Figure 2A,B). Analysis of the entire NGS panel revealed a *TP53* p.C242W (c.726C>G) in the neoplastic subarea, but not the two nonneoplastic subareas of case 10 (Figure 1C,D). The results indicated cytosine deamination artifact of case 10 leading to a false detection of *IDH1* c.395G>A. This specimen was excluded for further analysis of *IDH1*/*2*‐mutated NSCLCs.

**TABLE 1:** Coexisting mutations in lung adenocarcinomas with *IDH1*/*2* mutations

Cases	Tumor %^a^	7‐gene profiling^b^, ^c^	*IDH1*/*IDH*2^c^
1	21%‐40%	*KRAS* p.Q61H (10%)	*IDH1* p.R132H (25%, 558/741)
2	11%‐30%	*KRAS* p.G12D (7.9%)	*IDH1* p.R132L (11%, 938/8452)
3	51%‐70%	*EGFR* p.E746_A750del (33%)	*IDH1* p.R132H (3.0%, 174/5707)
4	71%‐90%	*KRAS* p.G12C (49%)	*IDH1* p.R132L (33%, 3360/10307)
5	31%‐50%	*PIK3CA* p.E542K (13%)	*IDH1* p.R132G (2.8%, 162/5841)
6	41%‐60%	*KRAS* p.G12V (18%)	*IDH1* p.R132L (3.8%, 290/7730)
7	41%‐60%	*KRAS* p.G12D (27%)	*IDH1* p.R132L (19%, 503/2649)
8	51%‐70%	*KRAS* p.G12D (34%)	*IDH2* p.R172S (13%, 282/2094)
9	41%‐60%	*KRAS* p.G12V (53%)	*IDH2* p.R140Q (13%, 260/2011)
			*IDH2* p.R172M (17%, 342/2022)
10^d^	11%‐30%	No mutation	*IDH1* p.R132H (4.6%, 109/2351)^d^

^a^ Estimated tumor cellularity.

^b^ *AKT1*, *BRAF*, *EGFR*, *ERBB2*, *KRAS*, *NRAS*, and *PIK3CA*.

^c^ Percentages and numbers in the parentheses indicate variant allele frequency and read depth of next‐generation sequencing. The numerator is the variant read number and the denominator is the total read number.

^d^ Quality assessment revealed c.395G>A (p.R132H) change resulting from cytosine deamination artifact. This specimen was excluded for further analysis.

*IDH1*/*2* mutations were detected in nine (0.5%) of 1924 patients with NSCLCs (Table 2). All nine patients were diagnosed as adenocarcinoma. There were three males and six females. The age ranged from 55 to 89 years with a median of 78 years. Seven (78%) of nine patients are 70 years or older. Six were current or former smokers and two were never smokers. Seven patients presented as a newly diagnosed lung cancer (3 with stage IA, 1 with stage IIIB, and 3 with stage IV). Case 9 revealed a residual lung adenocarcinoma, 18 months after the initial diagnosis of stage IIIB.

**TABLE 2:** Lung adenocarcinomas with *IDH1* or *IDH2* mutations

Cases	Age/gender	Smoking	Specimens	Stage^b^	HG^c^	TTF1/napsin A	PD‐L1
1	67/F	Yes	LLL (FNA)	IA	Yes	Positive/Positive	Not done
2	70/F	Yes	Lung, station 7 (FNA)	IIIB	Yes	Positive/Positive	Not done
3	55/F	No	RLL (Bx)	IA	Yes	Not done/Not done	Not done
4	78/M	No	LN, station 7 (FNA)	IV	Yes	Positive/Positive^a^	Not done
5	87/F	Yes	RUL (Bx)	IA	Yes	Positive/Not done	1%
6	89/M	Yes	Pleural effusion (TC)	IV	Yes	Positive/Positive	>95%
7	81/F	NK	Pleura (Bx)	NK	Yes	Positive/Not done	40%
8	83/F	Yes	Pleural effusion (TC)	IV	Yes	Positive/Positive	<1%
9	71/M	Yes	RUL (Bx)	IIIB	Yes	Positive/Positive^a^	85%

Abbreviations: Bx, biopsy; F, female; FNA, fine‐needle aspiration; LLL, left lower lobe; LN, lymph node; M, male; NK, not known; RLL, right lower lobe; RUL, right upper lobe; TC, thoracentesis.

^a^ Immunohistochemical stains were performed using another specimen taken from the same tumor.

^b^ Staging at initial diagnosis of lung cancer.

^c^ High‐grade (HG) histopathology or cytopathology.

All specimens had a coexisting driver mutation in another gene—seven specimens with a coexisting *KRAS* mutation, one with an *EGFR* mutation, and one with a *PIK3CA* mutation. *IDH1*/*2* mutations were not detected in *AKT1‐*, *BRAF‐*, *ERBB2‐,* or *NRAS*‐mutated NSCLCs. The incidence of *IDH1*/*2* mutations was significantly higher in *KRAS*‐mutated NSCLCs (n = 656) as compared with *KRAS* wild‐type NSCLCs (n = 1268) (1.1% vs 0.2%, *P* = .009). Six (86%) of seven patients with coexisting *KRAS* and *IDH1*/*2* mutations were 70 years or older, compared to 278 (43%) of 649 patients with only a *KRAS* mutation (*P* = .047).

### 3.2 Histomorphology and immunophenotypes of *IDH1/2*‐mutated lung adenocarcinomas

**FIGURE 3:**
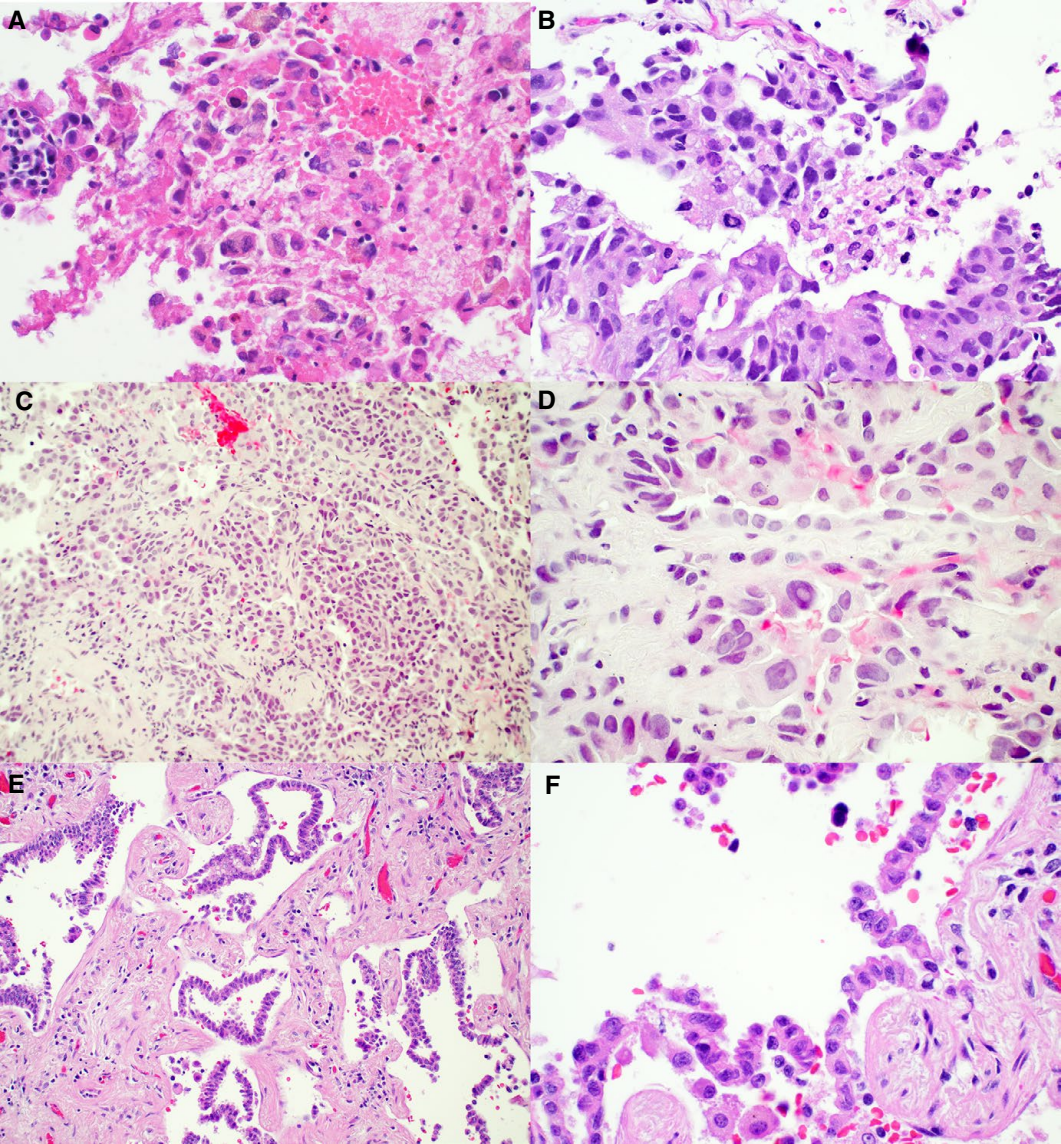
High‐grade features of IDH1/2 mutated lung adenocarcinomas (hematoxylin and eosin 600X for 3A, 3B, 3D and 3F and 200X for 3C and 3E). A, Cell block section (case 1 shows adenocarcinoma with marked pleomorphism and necrosis. B, Core biopsy of pleura (case 5) shows adenocarcinoma with solid growth pattern, marked pleomorphism, mitosis and necrosis. C‐D, High‐grade adenocarcinoma component of case 3 containing an IDH1 mutation. E‐F, well‐differentiated adenocarcinoma component of case 3 harboring an EGFR mutation

Specimens were taken by fine‐needle aspiration (FNA) of lung or lymph node (n = 3), thoracentesis of pleural effusion (n = 2), or core biopsy of lung or pleura (n = 4). Histomorphology of the core biopsy specimens and the cell blocks of FNA or thoracentesis specimens were reviewed by a cytopathologist (ER) and a pulmonary pathologist (PI). Necrosis was seen in three biopsy specimens and two FNA specimens. All five cytology specimens were characterized by high‐grade cytomorphology, such as high nuclear to cytoplasmic ratio, marked pleomorphism and/or prominent nucleoli (Figure 3A). All four biopsy specimens also showed high‐grade histopathology with marked pleomorphism (n = 4) and a predominantly solid pattern (n = 3) (Figure 3B). Extensive clear cell change, as described by Toth et al, was not observed in all nine adenocarcinomas.

Tumor cells were immunoreactive with TTF1 in eight of eight specimens examined and immunoreactive with Napsin A in six of six specimens examined, confirming a diagnosis of lung adenocarcinoma (Table 2). Immunohistochemical stains were not performed for case 3 taken from the right upper lobe and harboring an *EGFR* exon 19 deletion mutation, supporting a diagnosis of lung adenocarcinoma. High expression (50% or greater) of PD‐L1 was observed in two of five specimens examined.

### 3.3 Subclonal *IDH1/2* mutations

**FIGURE 4:**
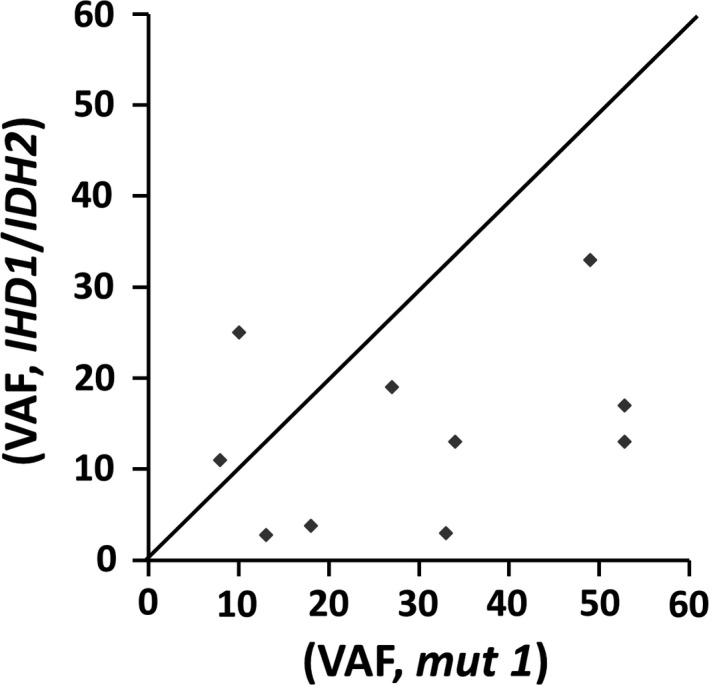
Correlation of variant allele frequency (VAF). VAF of IDH1/2 mutants vs VAF of KRAS (7 specimens), EGFR (one specimen) and PIK3CA (one specimen) mutants (mut 1 of the x‐axis), including case 9 with two IDH2 mutations

VAFs of *IDH1*/*2* mutants were higher than or concordant with those of *KRAS* mutants in cases 1 and 2, but were lower than those of *KRAS*, *EGFR,* or *PIK3CA* mutants in the remaining cases (Figure 4). The findings suggest a higher incidence of mutant allele–specific imbalance of the *KRAS*, *EGFR,* or *PIK3A* mutations or presence of subclonal tumor populations harboring *IDH1*/*2* mutations. The observations of less than 5% VAF of the *IDH1* mutation in cases 3, 5, and 6 in a contexts of 51%‐70%, 31%‐50%, and 41%‐60% estimated tumor cellularity and 33%, 13%, and 18% VAF of the coexisting *EGFR*, *KRAS,* or *PIK3CA* mutation suggest presence of *IDH1* mutation in a tumor subpopulation.

Subclonal evolution is also supported by examination of the resection specimen taken from case 3 at 2 months after biopsy. While NGS revealed 31% *EGFR* p.E746_A750del mutation and 3.0% *IDH1* p.R132H mutation in the biopsy specimen, the resection specimen showed the same *EGFR* mutation at 25%, but no *IDH1* mutation (Figure 2). Furthermore, the biopsy specimen showed a high‐grade features (Figure 3C,D), but the resection specimen showed a well‐differentiated adenocarcinoma with a lepidic growth pattern.

### 3.4 Parallel evolution of *IDH2* mutations

**FIGURE 5:**
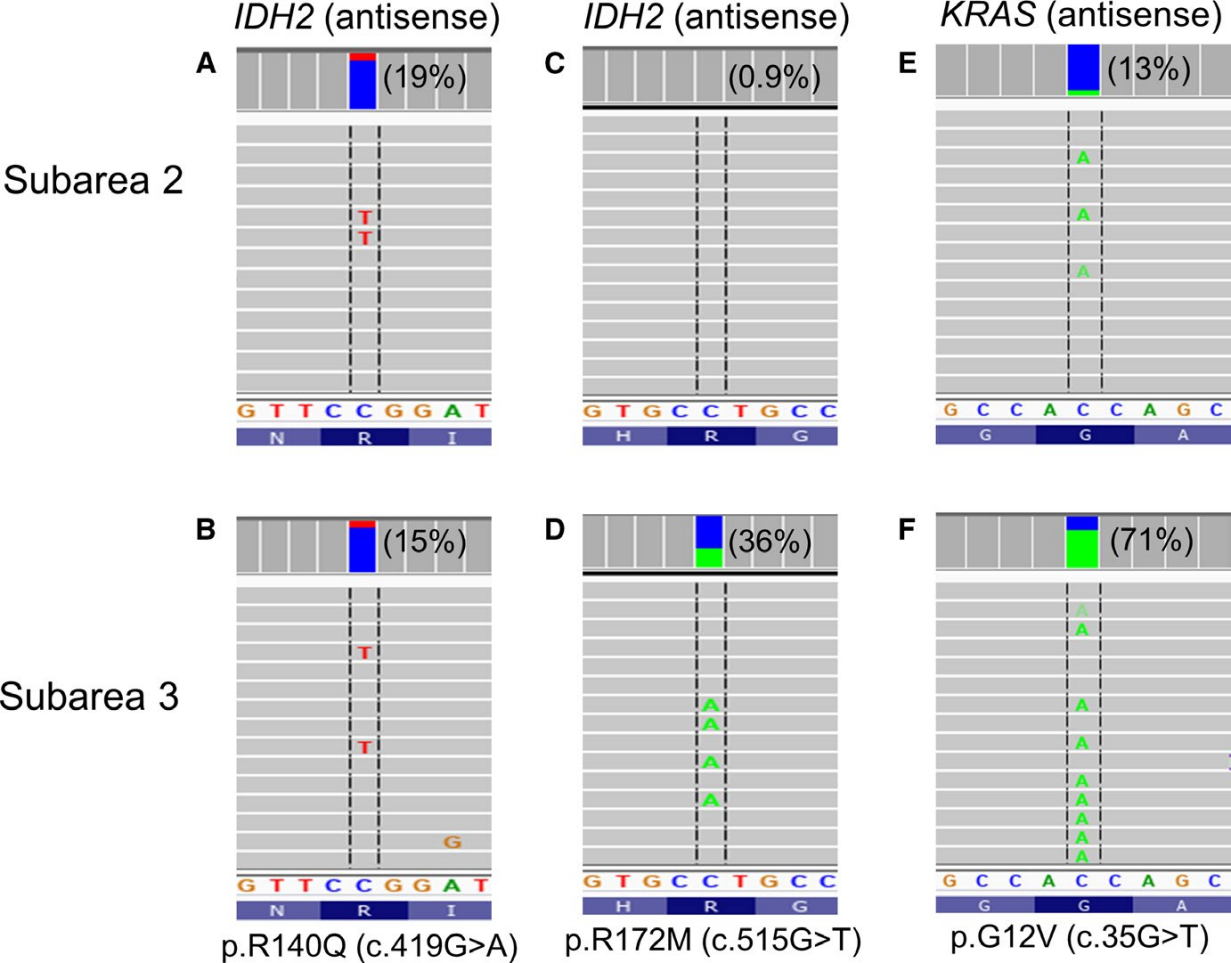
Multiregional analysis of KRAS mutated lung adenocarcinoma harboring two IDH2 mutations. In case 9, subarea 2 showed a dominant population with IDH2 p.R140Q and subarea 3 showed a dominant population with IDH2 p.R172M mutation. Percentage in parentheses indicates variant allele frequency

Case 9 was obtained 18 months after the initial diagnosis and treatment with chemotherapy and immune checkpoint blockage therapy (nivolumab). NGS analysis of this fragmented biopsy specimen containing approximately 41%‐60% estimated tumor cellularity revealed a *KRAS* mutation (VAF: 53%) and two *IDH2* mutations (VAFs: 13% for p.R140Q and 17% for p.R172M) within different alleles (Table 1). DNA was isolated from five randomly selected subareas showing similar histomorphology. The VAF ratio of p.R140Q and p.R172M was 21 (19% vs 0.9%) (Figure 5A,C), 6.8 (23% vs 3.4%), 2.3 (16% vs 6.9%), 0.99 (7.2% vs 7.3%), and 0.41 (15% vs 36%) (Figure 5B,D), respectively. Variation in VAF ratios from area to area support that the two *IDH2* mutations were present in different subpopulations harboring the same trunk *KRAS* driver mutation.

## 4 DISCUSSION

In this study with nearly 2000 NSCLC specimens, we confirmed a low incidence of *IDH1*/*2* mutations (0.5%), similar to those reported from MSK‐IMPACT (0.4%) and The Cancer Genome Atlas (TCGA) (0.4%) and relatively lower than that reported by Toth, et al (1.1%).^12^, ^13^, ^14^ The associations of *IDH1*/*2* mutations with smoking history (75%), 70 years or older (78%), and coexisting *KRAS* mutation (78%) are consistent with those previously observed (94%, 80%, and 69%, respectively).^12^, ^13^, ^14^ All 9 *IDH1/2‐*mutated lung adenocarcinomas showed high‐grade histopathology and/or cytopathology. Extensive clear cell change has been previously reported in lung adenocarcinomas with either *IDH1* p.R132C or p.R132L mutation,^14^ but not in nine lung adenocarcinomas in this study, including four with an *IDH1* p.R132L mutation.

Tumor heterogeneity has important clinical applications in targeted therapy.^23^, ^24^ Trunk (initiating) driver mutations initiate the development of a founding cancer cell. Subsequently, branching driver mutations promote subclonal evolution. Therefore, trunk drivers are present in each cancer cell of the primary and metastatic tumors, while branching drivers may be present in a dominant subpopulation or a minor subclone. Targeting a mutation present only in a minor subclone likely results in a shorter progression‐free survival, even though the initial efficacy is observed. A combinatory therapy to include trunk drivers, if present, for targeting may provide better treatment outcomes. We have previously shown that lower than expected VAF indicates intratumor heterogeneity while higher than expected VAF indicates mutant allele–specific imbalance.^25^ In this study, the high incidence of coexisting *KRAS* and *EGFR* driver mutations and the lower than expected *IDH1*/*2* VAF suggest that *IDH1*/*2* mutations may be branching drivers promoting subclonal progression of lung adenocarcinomas.

By tracking the evolution of lung adenocarcinomas, most *BRAF*, *EGFR,* and *KRAS* activating mutations are trunk drivers.^26^ These trunk drivers are usually mutually exclusive, although rare cases with coexisting *EGFR* and *KRAS* mutations have been reported.^27^ In contrast, *TP53* and *PIK3CA* mutations are branching drivers and can be seen in *BRAF‐*, *EFGR‐*, or *KRAS*‐mutated lung adenocarcinomas. In a total of 25 *IDH1/2*‐mutated lung adenocarcinomas reported by Toth et al, TCGA, MSK‐IMPACT, and this study, known trunk drivers were seen in 23 cases.^14^ These included 18 cases with a *KRAS* mutation, four cases with an *EGFR* mutation, and one case with *BRAF* p.V600E. Coexistence of *IDH1/2* mutations with trunk drivers within different genes suggests *IDH1/2* mutations are branching drivers. In case 3 from our cohort, the observation of an *EGFR* mutation in both the biopsy and resection specimens of the same tumor, but the presence of *IDH1* mutation only in the biopsy specimen also supports that the *IDH1* mutation was a branching driver occurring during the subclonal evolution in lung adenocarcinoma. Immunohistochemical stains could also be helpful to identify tumor heterogeneity of the IDH1 p.R132H mutant.

Mutations within the same signature transduction pathway are redundant and, therefore, most mutually exclusive. Detection of coexisting mutations within the same pathway raises the concern for potential laboratory errors.^27^ In a previous study for quality assessment, we confirmed that *KRAS* and *NRAS* mutations may be present in the same population or different subpopulations of colorectal cancers according to an operating procedure proposed for validation of unexpected coexisting mutations.^28^ Concurrent *IDH1* and *IDH2* mutations have been reported in two AMLs,^5^ four anaplastic gliomas,^8^ and three chondrosarcomas.^29^ Whether multiple *IDH1*/*2* mutations occurred in the same or different tumor subpopulations was not described. In this study, multiregional analyses of case 9 support the presence of two distinct *IDH2* mutations in different subpopulations harboring the same trunk driver *KRAS* mutation.

Parallel evolution in neoplasms refers to evolution of distinct subpopulations from a common ancestral clone as a consequence of independent mutations affecting the same gene or genes in the same pathway.^30^, ^31^ Parallel evolution has been shown in a variety of neoplasms, including lung cancers involving genetic alterations in the *MUC1*, *CDK4*, *CHD8,* and *NKX2‐1* genes.^26^, ^30^, ^31^ Results of multiregional analyses in case 9 indicate paralleled evolution involving the *IDH2* gene and support subclonal evolution promoted by two *IDH2* mutations as branching drivers.

Previous studies have confirmed the role of *IDH1* and *IDH2* driver mutations in tumorigenesis of AML and gliomas.^16^, ^32^, ^33^ In vitro studies showed *IDH1* p.R132H mutation enhances migration and proliferation of NSCLC cells through downregulation of fibulin 5 by hypermethylation of the promoter.^34^ In lung cancers, plasma IDH1 and IDH2 proteins are elevated and could be novel biomarkers for diagnosis of NSCLCs.^35^, ^36^ A germline variant (rs11540478) within the *IDH2* gene is associated with an increased risk of lung cancers.^37^ Cannataro et al have proposed a novel concept, the cancer effect size of genetic alterations, to estimate the intensity of selective advantage to cancer cells.^38^ As expected, mutations of *IDH1* codon 132 show a very high effect size (greater than 10^6^ or 10^7^) in lower‐grade glioma of the brain. *IDH1* p.R132C also shows a relatively higher effect size (10^4^‐10^5^) in lung adenocarcinomas. These findings suggest *IDH1/2* mutations may confer selective advantage for clonal evolution in lung adenocarcinomas.

*IDH1* and *IDH2* mutations are detected in approximately 60%‐80% and 1%‐5% of patients with WHO grades II‐III astrocytoma or oligodendroglioma and are predominantly *IDH1* p.R132H (>90%).^8^ In gliomas of the brain, *IDH1/2* mutations are trunk drivers to confer cancer initiation and are favorable prognostic markers.^8^, ^16^, ^39^, ^40^ Combined analysis of 26 *IDH1*/*2* mutations in NSCLCs reported in the previous and current studies revealed predominantly *IDH1* p.R132C (35%) and p.R132L (27%). *IDH1* p.R132H was seen in only two cases (7.7%). In this study, *IDH1/2* mutations occurred in adenocarcinomas with high‐grade features and may be branching drivers leading to subclonal evolution. Further prospective studies in a larger cohort are warranted to elucidate if *IDH1/2* mutations are markers for theranostics and/or prognostication in lung cancers.

We and others have shown that cytosine deamination changes induced by formalin fixation or thermocycling of polymerase chain reaction is a major cause of background noise in both Sanger sequencing and NGS.^21^, ^41^, ^42^ Pretreatment of DNA specimens with uracil‐*N*‐glycosylase can reduce the level of cytosine deamination artifact.^42^, ^43^ During our validation of this NGS panel, we have shown that background noise is consistent with spontaneous and formalin‐induced cytosine deamination change.^21^ Therefore, a cutoff of background noise at 2% was chosen for single‐nucleotide variations. In our daily routine practice, background noise resulting from cytosine deamination changes is often less 2%. However, up to 2%‐5% of cytosine deamination artifact occurring at multiple‐nucleotide positions can be observed when the DNA input is suboptimal (usually less than 10 ng), consistent with previous observations that the chance of cytosine deamination artifact was inversely correlated with input of DNA quantity for sequencing.^41^ Repeat of NGS often shows a low level of cytosine deamination changes occurring at other nucleotide positions. The observation of *IDH1* c.359G>A (p.R132H) in case 10 was uncommon for cytosine deamination artifacts. In this specimen, cytosine deamination changes with frequency of 2% or more at other nucleotide positions were not observed and repeat of NGS showed persistent *IDH1* c.359G>A change. However, NGS analyses of two subareas containing no tumor cells also show c.359G>A change indicating a false detection of *IDH1* c.395G>A.

Although the incidence of *IDH1*/*2* mutations is only 0.4%‐1.1% in NSCLCs, it is worthwhile to investigate IDH1/2 inhibitors because of the high prevalence of lung cancers in the United States and worldwide. On the other hand, *IDH1*/*2* mutations may be branching drivers leading to subclonal evolution, which may affect the benefit of IDH1/2 inhibitors. Accumulation of more *IDH1*/*2*‐mutated NSCLCs is needed to elucidate their clinicopathological characteristics and implications for targeted therapy.

## CONFLICT OF INTEREST

All authors declare no conflict of interest.

## AUTHORS’ CONTRIBUTION

Erika Rodriguez and Federico De Marchi (First authors): conceptualization, methodology, validation, formal analysis, investigation, resources, data curation, and writing original draft. Parvez M. Lokhandwala, Deborah Belchis, Rena Xian, Christopher D. Gocke, James R. Eshleman, and Peter Illei: investigation, resources, data curation, and editing manuscript. Ming‐Tseh Li: conceptualization, methodology, validation, formal analysis, investigation, resources, data curation, writing review and editing, visualization, supervision, and project administration.

## Data Availability

The data that support the findings of this study are available from the corresponding author upon reasonable request.

## References

[1] Reck M , Rabe KF . Precision diagnosis and treatment for advanced non‐small‐cell lung cancer. N Engl J Med. 2017;377:849‐861. 28854088 10.1056/NEJMra1703413

[2] Brown NA , Aisner DL , Oxnard GR . Precision medicine in non‐small cell lung cancer: current standards in pathology and biomarker interpretation. Am Soc Clin Oncol Educ Book. 2018;38:708‐715. 30231309 10.1200/EDBK_209089

[3] Lindeman NI , Cagle PT , Aisner DL , et al. Updated molecular testing guideline for the selection of lung cancer patients for treatment with targeted tyrosine kinase inhibitors: guideline from the College of American Pathologists, the International Association for the Study of Lung Cancer, and the Association for Molecular Pathology. Arch Pathol Lab Med. 2018;142:321‐346. 29355391 10.5858/arpa.2017-0388-CP

[4] Marcucci G , Maharry K , Wu YZ , et al. IDH1 and IDH2 gene mutations identify novel molecular subsets within de novo cytogenetically normal acute myeloid leukemia: a Cancer and Leukemia Group B study. J Clin Oncol. 2010;28:2348‐2355. 20368543 10.1200/JCO.2009.27.3730 PMC2881719

[5] Paschka P , Schlenk RF , Gaidzik VI , et al. IDH1 and IDH2 mutations are frequent genetic alterations in acute myeloid leukemia and confer adverse prognosis in cytogenetically normal acute myeloid leukemia with NPM1 mutation without FLT3 internal tandem duplication. J Clin Oncol. 2010;28:3636‐3643. 20567020 10.1200/JCO.2010.28.3762

[6] Stein EM , DiNardo CD , Pollyea DA , et al. Enasidenib in mutant IDH2 relapsed or refractory acute myeloid leukemia. Blood. 2017;130:722‐731. 28588020 10.1182/blood-2017-04-779405 PMC5572791

[7] DiNardo CD , Stein EM , de Botton S , et al. Durable remissions with Ivosidenib in IDH1‐mutated relapsed or refractory AML. N Engl J Med. 2018;378:2386‐2398. 29860938 10.1056/NEJMoa1716984

[8] Hartmann C , Meyer J , Balss J , et al. Type and frequency of IDH1 and IDH2 mutations are related to astrocytic and oligodendroglial differentiation and age: a study of 1,010 diffuse gliomas. Acta Neuropathol. 2009;118:469‐474. 19554337 10.1007/s00401-009-0561-9

[9] Borger DR , Tanabe KK , Fan KC , et al. Frequent mutation of isocitrate dehydrogenase (IDH)1 and IDH2 in cholangiocarcinoma identified through broad‐based tumor genotyping. Oncologist. 2012;17:72‐79. 22180306 10.1634/theoncologist.2011-0386 PMC3267826

[10] Kipp BR , Voss JS , Kerr SE , et al. Isocitrate dehydrogenase 1 and 2 mutations in cholangiocarcinoma. Hum Pathol. 2012;43:1552‐1558. 22503487 10.1016/j.humpath.2011.12.007

[11] Amary MF , Bacsi K , Maggiani F , et al. IDH1 and IDH2 mutations are frequent events in central chondrosarcoma and central and periosteal chondromas but not in other mesenchymal tumours. J Pathol. 2011;224:334‐343. 21598255 10.1002/path.2913

[12] Gutman DA , Cobb J , Somanna D , et al. Cancer digital slide archive: an informatics resource to support integrated in silico analysis of TCGA pathology data. J Am Med Inform Assoc. 2013;20:1091‐1098. 23893318 10.1136/amiajnl-2012-001469 PMC3822112

[13] Zehir A , Benayed R , Shah RH , et al. Mutational landscape of metastatic cancer revealed from prospective clinical sequencing of 10,000 patients. Nat Med. 2017;23:703‐713. 28481359 10.1038/nm.4333 PMC5461196

[14] Toth LN , de Abreu FB , Tafe LJ . Non‐small cell lung cancers with isocitrate dehydrogenase 1 or 2 (IDH1/2) mutations. Hum Pathol. 2018;78:138‐143. 29723602 10.1016/j.humpath.2018.04.014

[15] Dang L , White DW , Gross S , et al. Cancer‐associated IDH1 mutations produce 2‐hydroxyglutarate. Nature. 2010;465:966. 20559394 10.1038/nature09132 PMC3766976

[16] Molenaar RJ , Radivoyevitch T , Maciejewski JP , van Noorden CJ , Bleeker FE . The driver and passenger effects of isocitrate dehydrogenase 1 and 2 mutations in oncogenesis and survival prolongation. Biochim Biophys Acta. 2014;1846:326‐341. 24880135 10.1016/j.bbcan.2014.05.004

[17] Fan B , Mellinghoff IK , Wen PY , et al. Clinical pharmacokinetics and pharmacodynamics of ivosidenib, an oral, targeted inhibitor of mutant IDH1, in patients with advanced solid tumors. Invest New Drugs. 2020;38(2):433‐444. 31028664 10.1007/s10637-019-00771-x PMC7066280

[18] Zheng G , Lin M‐T , Lokhandwala PM , et al. Clinical mutational profiling of bone metastases of lung and colon carcinoma and malignant melanoma using next‐generation sequencing. Cancer Cytopathol. 2016;124:744‐753. 27286239 10.1002/cncy.21743

[19] Illei PB , Belchis D , Tseng L‐H , et al. Clinical mutational profiling of 1006 lung cancers by next generation sequencing. Oncotarget. 2017;8:96684‐96696. 29228562 10.18632/oncotarget.18042 PMC5722514

[20] Thorvaldsdóttir H , Robinson JT , Mesirov JP . Integrative Genomics Viewer (IGV): high‐performance genomics data visualization and exploration. Brief Bioinform. 2013;14:178‐192. 22517427 10.1093/bib/bbs017 PMC3603213

[21] Lin M‐T , Mosier SL , Thiess M , et al. Clinical validation of KRAS, BRAF, and EGFR mutation detection using next‐generation sequencing. Am J Clin Pathol. 2014;141:856‐866. 24838331 10.1309/AJCPMWGWGO34EGOD PMC4332779

[22] Cowan ML , Li QK , Illei PB . CDX‐2 expression in primary lung adenocarcinoma. Appl Immunohistochem Mol Morphol. 2016;24:16‐19. 26469326 10.1097/PAI.0000000000000250

[23] Gerlinger M , Rowan AJ , Horswell S , et al. Intratumor heterogeneity and branched evolution revealed by multiregion sequencing. N Engl J Med. 2012;366:883‐892. 22397650 10.1056/NEJMoa1113205 PMC4878653

[24] Vogelstein B , Papadopoulos N , Velculescu VE , Zhou S , Diaz LA Jr , Kinzler KW . Cancer genome landscapes. Science. 2013;339:1546‐1558. 23539594 10.1126/science.1235122 PMC3749880

[25] Haley L , Tseng L‐H , Zheng G , et al. Performance characteristics of next‐generation sequencing in clinical mutation detection of colorectal cancers. Mod Pathol. 2015;28:1390‐1399. 26226847 10.1038/modpathol.2015.86 PMC4618462

[26] Jamal‐Hanjani M , Wilson GA , McGranahan N , et al. Tracking the evolution of non‐small‐cell lung cancer. N Engl J Med. 2017;376:2109‐2121. 28445112 10.1056/NEJMoa1616288

[27] De Marchi F , Haley L , Fryer H , et al. Clinical validation of coexisting activating mutations within EGFR, mitogen‐activated protein kinase, and phosphatidylinositol 3‐kinase pathways in lung cancers. Arch Pathol Lab Med. 2019;143:174‐182. 30485130 10.5858/arpa.2017-0495-OA

[28] Tseng LH , De Marchi F , Pallavajjalla A , et al. Clinical validation of discordant trunk driver mutations in paired primary and metastatic lung cancer specimens. Am J Clin Pathol. 2019;152(5):570‐581. 31264684 10.1093/ajcp/aqz077 PMC6779251

[29] Lugowska I , Teterycz P , Mikula M , et al. IDH1/2 mutations predict shorter survival in chondrosarcoma. J Cancer. 2018;9:998‐1005. 29581779 10.7150/jca.22915 PMC5868167

[30] McGranahan N , Favero F , de Bruin EC , Birkbak NJ , Szallasi Z , Swanton C . Clonal status of actionable driver events and the timing of mutational processes in cancer evolution. Sci Transl Med. 2015;7:283ra54. 10.1126/scitranslmed.aaa1408 PMC4636056 25877892

[31] McGranahan N , Swanton C . Clonal heterogeneity and tumor evolution: past, present, and the future. Cell. 2017;168:613‐628. 28187284 10.1016/j.cell.2017.01.018

[32] Medeiros BC , Fathi AT , DiNardo CD , Pollyea DA , Chan SM , Swords R . Isocitrate dehydrogenase mutations in myeloid malignancies. Leukemia. 2017;31:272‐281. 27721426 10.1038/leu.2016.275 PMC5292675

[33] Dang L , Yen K , Attar EC . IDH mutations in cancer and progress toward development of targeted therapeutics. Ann Oncol. 2016;27:599‐608. 27005468 10.1093/annonc/mdw013

[34] Yan B , Hu Y , Ma T , Wang Y . IDH1 mutation promotes lung cancer cell proliferation through methylation of Fibulin‐5. Open Biol. 2018;8:180086. 30305430 10.1098/rsob.180086 PMC6223204

[35] Sun N , Chen Z , Tan F , et al. Isocitrate dehydrogenase 1 is a novel plasma biomarker for the diagnosis of non‐small cell lung cancer. Clin Cancer Res. 2013;19:5136‐5145. 24046070 10.1158/1078-0432.CCR-13-0046

[36] Li J‐J , Li R , Wang W , et al. IDH2 is a novel diagnostic and prognostic serum biomarker for non‐small‐cell lung cancer. Mol Oncol. 2018;12:602‐610. 29465809 10.1002/1878-0261.12182 PMC5928355

[37] Li J , Lu J , He Y , et al. A new functional IDH2 genetic variant is associated with the risk of lung cancer. Mol Carcinog. 2017;56:1082‐1087. 27649069 10.1002/mc.22573

[38] Cannataro VL , Gaffney SG , Townsend JP . Effect sizes of somatic mutations in cancer. J Natl Cancer Inst. 2018;110:1171‐1177. 30365005 10.1093/jnci/djy168 PMC6235682

[39] Sanson M , Marie Y , Paris S , et al. Isocitrate dehydrogenase 1 codon 132 mutation is an important prognostic biomarker in gliomas. J Clin Oncol. 2009;27:4150‐4154. 19636000 10.1200/JCO.2009.21.9832

[40] Xia L , Wu B , Fu Z , et al. Prognostic role of IDH mutations in gliomas: a meta‐analysis of 55 observational studies. Oncotarget. 2015;6:17354‐17365. 26220714 10.18632/oncotarget.4008 PMC4627313

[41] Williams C , Pontén F , Moberg C , et al. A high frequency of sequence alterations is due to formalin fixation of archival specimens. Am J Pathol. 1999;155:1467‐1471. 10550302 10.1016/S0002-9440(10)65461-2 PMC1866966

[42] Chen G , Mosier S , Gocke CD , Lin MT , Eshleman JR . Cytosine deamination is a major cause of baseline noise in next‐generation sequencing. Mol Diagn Ther. 2014;18:587‐593. 25091469 10.1007/s40291-014-0115-2 PMC4175022

[43] Do H , Dobrovic A . Dramatic reduction of sequence artefacts from DNA isolated from formalin‐fixed cancer biopsies by treatment with uracil‐ DNA glycosylase. Oncotarget. 2012;3:546‐558. 22643842 10.18632/oncotarget.503 PMC3388184

